# Sensitivity of *Clarireedia* spp. to benzimidazoles and dimethyl inhibitors fungicides and efficacy of biofungicides on dollar spot of warm season turfgrass

**DOI:** 10.3389/fpls.2023.1155670

**Published:** 2023-06-09

**Authors:** Bikash Ghimire, Md. Aktaruzzaman, Shukti R. Chowdhury, Willis T. Spratling, C. Brian Vermeer, James W. Buck, Alfredo D. Martinez-Espinoza, Bochra A. Bahri

**Affiliations:** ^1^ Department of Plant Pathology, University of Georgia, Griffin, GA, United States; ^2^ Institute of Plant Breeding, Genetics and Genomics, University of Georgia, Griffin, GA, United States; ^3^ Department of Plant Pathology, University of Georgia, Tifton, GA, United States; ^4^ Department of Plant Pathology, Sher-e-Bangla Agricultural University, Dhaka, Bangladesh

**Keywords:** turfgrass, *Clarireedia*, dollar spot, fungicide resistance, biofungicide, thiophanate-methyl, propiconazole, *Bacillus subtilis* QST713

## Abstract

Dollar spot caused by *Clarireedia* spp. (formerly *Sclerotinia homoeocarpa*) is an economically destructive fungal disease of turfgrass that can significantly compromise turf quality, playability, and aesthetic value. Fungicides are frequently used to manage the disease but are costly and potentially unfavorable to the environment. Repeated use of some active ingredients has resulted in reduced efficacy on *C. jacksonii* causing dollar spot in cool-season turfgrasses in the US. Experiments were conducted to study fungicide sensitivity of *Clarireedia* spp. as well as to develop alternatives to fungicides against dollar spot on warm-season turfgrass in Georgia. First, 79 isolates of *Clarireedia* spp. collected across the state were tested *on* fungicide-amended agar plates for their sensitivity to thiophanate-methyl (benzimidazole) and propiconazole (dimethyl inhibitor). Seventy-seven isolates (97.5%) were sensitive (0.001 to 0.654 μg/mL) and two isolates (2.5%) were found resistant (>1000 μg/mL) to thiophanate-methyl. However, in the case of propiconazole, 27 isolates (34.2%) were sensitive (0.005 to 0.098 μg/mL) while 52 isolates (65.8%) were resistant (0.101 to 3.820 μg/mL). Next, the efficacy of three bio- and six synthetic fungicides and ten different combinations were tested *in vitro* against *C. monteithiana*. Seven bio- and synthetic fungicide spray programs comprising *Bacillus subtilis* QST713 and propiconazole were further tested, either alone or in a tank mix in a reduced rate, on dollar spot infected bermudagrass ‘TifTuf’ in growth chamber and field environments. These fungicides were selected as they were found to significantly reduce pathogen growth up to 100% on *in vitro* assays. The most effective spray program in growth chamber assays was 100% *B. subtilis* QST713 in rotation with 75% *B. subtilis* QST713 + 25% propiconazole tank mix applied every 14 days. However, the stand-alone application of the biofungicide *B. subtilis* QST713 every seven days was an effective alternative and equally efficacious as propiconazole, suppressing dollar spot severity and AUDPC up to 75%, while resulting in acceptable turf quality (>7.0) in field experiments. Our study suggests that increased resistance of *Clarireedia* spp. to benzimidazoles and dimethyl inhibitors warrants continuous surveillance and that biofungicides hold promise to complement synthetic fungicides in an efficacious and environmentally friendly disease management program.

## 1 Introduction

The turfgrass industry is extended across the globe and contributes nearly $84 billion to the United States economy (National Golf Foundation (NGF), 2016). It is estimated that there are approximately 62 million acres of turfgrass in the United States alone, making it the fourth-largest crop in acreage (Chawla et al., 2018). Dollar spot is among the most commercially significant turfgrass diseases in the world (Vargas, 2018). At present, six species within the genus *Clarireedia* are known to be responsible for causing dollar spot in turfgrasses (Salgado-Salazar et al., 2018; Hu et al., 2019; Hu et al., 2021; Zhang et al., 2022). Among them, *C. jacksonii* and *C. monteithiana* are the two most prominent species found in the US (Salgado-Salazar et al., 2018; Hu et al., 2019). Previous studies showed host specificity where *C. jacksonii* is mostly present in cool-season turfgrasses while *C. monteithiana* predominates warm-season turfgrasses (Salgado-Salazar et al, 2018).

Management of dollar spot highly relies on the use of fungicides which are costly and potentially detrimental to the environment (Knopper and Lean, 2010). In the US and worldwide, a high economic burden incurs on the chemical control of this disease requiring more than ten applications for the growing season (Allen et al., 2005; Koch et al., 2021; Sapkota et al., 2022). The cost of fungicide application to manage dollar spot in a single golf course lies between $10,000-$34,000 annually (Golf Course Industry, 2015; Bekken et al., 2022). Four main fungicide groups are widely utilized across the globe for the management of dollar spot which include dicarboximides (DCFs), demethylase inhibitors (DMIs), succinate dehydrogenase inhibitors (SDHIs), and methyl benzimidazole carbamates (MBCs) (FRAC, https://www.frac.info/home ) (Ok et al., 2011; Popko et al., 2012; Zhang et al., 2021). However, repeated applications of fungicides favored isolates of *Clarireedia* species with decreased sensitivity to fungicides with different modes of action (Sang et al., 2015; Jian et al., 2017; Popko et al., 2018). To date, five *Clarireedia* species (except the newly identified species *C. hainanense*) resistant to all four fungicide groups can be found worldwide (Zhang et al., 2021). Resistance of dollar spot pathogen populations to MBC and DCF fungicides was observed shortly after their introduction in the early 1970s (Warren et al., 1974) and reduced sensitivity to DMI fungicide was reported after a decade of use in Canada (Hsiang et al., 2007). For instance, Zhang et al. (2021) assessed 358 isolates of *Clarireedia* spp. collected from creeping bentgrass and seashore paspalum across 25 locations in China and revealed that 81, 67, 63, and 62% of these isolates had reduced sensitivity to propiconazole, boscalid (SDHI), iprodione, and thiophanate-methyl, respectively. In Putman et al. (2010), twenty populations comprising a total of 965 isolates of *Clarireedia* spp. collected from cool-season turf grasses in the northeast US had fourteen and eighteen populations with reduced sensitivity to iprodione (DCF) and propiconazole (DMI), respectively. For thiophanate-methyl (MBC), five populations were sensitive, while nine populations contained varying proportions (2 to 92%) of resistant isolates (Putman et al., 2010). Recently, Popko et al. (2018) documented SDHI-resistant *Clarireedia* strains in Japan and cool-season turfgrass in the northeast US in 2016 and 2017, respectively. More importantly, there is an increased concern due to the emergence of multiple fungicide resistant (MFR) *Clarireedia* populations in cool-season turfgrass in the northeast US (Putman et al., 2010; Stephens and Kaminski, 2019). So far, there is only a single report on the fungicide sensitivity of dollar spot isolates to propiconazole collected from creeping bentgrass in Georgia, USA (Miller et al., 2002). Therefore, routine monitoring and surveillance of these fungal isolates is indispensable in the US, including in the state of Georgia where fungicides are continuously applied on turfgrass.

The widespread use of fungicides and the subsequent rise in the prevalence of fungicide resistance in target populations has prompted a search for non-chemical alternatives to manage the disease. One alternative is biological control agents (BCAs) that manage population levels of a pathogen, through either antibiosis, competition, mycoparasitism or to reduce the incidence of plant diseases via metabolite production. A biofungicide either alone or in a tank mix with synthetic fungicides could reduce the reliance on chemicals for controlling dollar spot. At present, biocontrol products such as Rhapsody™ (*Bacillus subtilis* QST713), and Regalia™ (plant extract of *Reynoutria sachalinensis*, hereafter termed as *R. sachalinensis* extr.) are registered for the control of dollar spot (Allen et al., 2005). Clarke et al. (2020) listed *B. licheniformis* (Ecoguard™), *B. subtilis* QST713, and *R. sachalinensis* extr. as potential biological control agents (BCAs) to control dollar spot by reviewing more than 1000 research reports published over a 38-year period in Plant Disease Management Reports. Previous studies showed the successful inclusion of several BCAs against *C. jacksonii* including *Enterobacter cloacae* strains EcCT-501 on creeping bentgrass/annual bluegrass putting greens in New York (Nelson and Craft, 1991) and *Pseudomonas aureofaciens* Tx-1 on creeping bentgrass in Michigan (Powell et al., 2000). Marvin et al. (2020a) evaluated the curative control efficacy of four BCAs (*B. licheniformis* SB3086, *B. subtilis* QST713, *R. sachalinensis* extr., and plant extract oils) along with two synthetic fungicides (chlorothalonil and azoxystrobin + propiconazole) in a different tank-mix combination and reported that all BCAs except *R. sachalinensis* extr. provided acceptable disease control (disease severity ≤15%) and turfgrass quality (≥7) against *C. jacksonii* in South Carolina on creeping bentgrass putting greens. Recently, secondary metabolites derived from *Streptomyces* spp. AN090126 showed promising results on *in vitro* and pot experiments carried out in creeping bentgrass in Korea signifying its potential as a BCA (Le et al., 2022). Provided that most of the studies were carried out on cool-season turf and/or against *C. jacksonii* in the northeast US, there is an information gap for fungicide sensitivity of *Clarireedia* populations and efficacy of biofungicides against dollar spot in the southeastern state of Georgia where commonly grown warm-season turfgrass is mostly challenged by *C. monteithiana*.

This study was conducted with the objectives to assess fungicide sensitivity of *Clarireedia* spp. in Georgia and to develop efficient alternative biofungicide programs to effectively manage dollar spot. In this study, we report *in vitro* sensitivities to thiophanate-methyl and propiconazole for 79 isolates of *Clarireedia* spp. collected from 42 counties in Georgia as well as the efficacy of three biofungicides against six synthetic fungicides and their combinations *in vitro*, in growth chambers, and in the field.

## 2 Materials and methods

### 2.1 Sample collection, fungal isolation, and inoculum preparation

A total of 79 *Clarireedia* spp. isolates collected from 42 counties throughout the state of Georgia, US from 2019-2022 were used in this study. The collection was conducted on several warm-season turfgrass species and included athletic fields, golf courses, homeowner, landscape, University grounds and research areas (**Figure S1**, **Table 1**). *Clarireedia* spp. was isolated from symptomatic leaf tissue and pure cultures of the pathogen were obtained using several hyphal tip transfers. *Clarireedia* spp. were confirmed by the colony and hyphal morphology and Sanger sequencing of the internal transcribed spacer (ITS4 and ITS5) region, as described by Sapkota et al. (2020). Among 79 isolates, 75 isolates were *C. monteithiana* and four isolates were *C. jacksonii*. Ten agar plugs (5 mm in diameter) of each isolate were placed in a 2 mL microtube, immersed in 20% glycerin, and stored short-term at 4°C until further use. Each isolate was also long-term stored on oat/barley/wheat grain mixture at -20°C (Steketee et al., 2016). *C. monteithiana* DS8 was used in the biofungicide efficacy experiments. The isolate was collected in 2019 at the University of Georgia (UGA) Griffin Campus from seashore paspalum (ITS sequence stored in the NCBI GenBank under ‘MT497854’). For *in vitro* biofungicide experiments, *C. monteithiana* DS8 was grown on potato dextrose agar (PDA) for five days under 12-hour light at room temperature before the experiments were initiated. For growth chamber and field experiments, the isolate was grown in 250 ml Erlenmeyer flasks on an oat/barley/wheat grain mixture previously soaked in water overnight and double sterilized (Steketee et al., 2016). Briefly, a total of 100 g grain mixture was soaked in 100 ml water and 20 agar plugs (7 mm diameter) of *C. monteithiana* DS8 were placed in the sterile grain flask and cultured for 7 days at room temperature with a 12-hour photoperiod.

**Table 1:** *Clarireedia* spp. isolates, isolated host, isolation year, season, and location used in this study along with their EC_50_ value for thiophanate-methyl and propiconazole assessed *in vitro*.

Isolate name	Host^a^	Season	Date Isolated	Location	Categories	Causal organism	EC_50_ value (μg/mL)			
							Thiophanate-methyl		Propiconazole	
							Mean ± SD	Resistant Reaction^b^	Mean ± SD	Resistant Reaction^c^
**DS7**	*Zoysia* sp.	Fall	2019	Spalding	University grounds	*C. monteithiana*	0.010 ± 0.0007	HS	0.041 ± 0.025	MS
**DS8**	*Paspalum vaginatum* Swartz	Fall	2019	Spalding	University grounds	*C. monteithiana*	0.025 ± 0.002	MS	0.131 ± 0.100	LR
**DS3**	*Agrostis stolonifera* L.	Fall	2019	Spalding	University grounds	*C. jacksonii*	>1000	HR	0.15 ± 0.03	LR
**DS9**	*Cynodon dactylon* L.	Fall	2019	Spalding	University grounds	*C. monteithiana*	0.034 ± 0.01	MS	0.042 ± 0.003	MS
**DS1**	*Zoysia* sp.	Fall	2020	Clarke	Homeowner	*C. monteithiana*	0.006 ± 0.003	HS	0.07 ± 0.02	LS
**DS2**	*Zoysia* sp.	Fall	2020	Bibb	Homeowner	*C. monteithiana*	0.027 ± 0.005	MS	0.41 ± 0.16	MR
**DS4**	*Zoysia* sp.	Fall	2020	Spalding	Landscape	*C. monteithiana*	0.014 ± 0.005	MS	0.27 ± 0.06	MR
**DS179**	*Zoysia* sp.	Fall	2020	Clarke	Homeowner	*C. monteithiana*	0.052 ± 0.003	MS	0.383 ± 0.128	MR
**DS180**	*Zoysia* sp.	Summer	2020	Spalding	University grounds	*C. monteithiana*	0.004 ± 0.0006	HS	0.355 ± 0.035	MR
**DS181**	*Zoysia* sp.	Summer	2020	Fulton	Homeowner	*C. monteithiana*	0.005 ± 0.0006	HS	0.153 ± 0.045	LR
**DS182**	*Cynodon dactylon* L.	Summer	2020	Cook	Landscape	*C. monteithiana*	0.137 ± 0.02	LS	0.104 ± 0.009	LR
**DS183**	*Paspalum vaginatum* Swartz	Summer	2020	Cook	Homeowner	*C. monteithiana*	0.072 ± 0.006	MS	0.143 ± 0.010	LR
**DS184**	*Cynodon dactylon* L.	Summer	2020	Spalding	University grounds	*C. monteithiana*	0.302 ± 0.04	LS	0.271 ± 0.023	MR
**DS185**	*Digitaria* sp.	Fall	2020	Spalding	Landscape	*C. jacksonii*	0.003 ± 0.0007	HS	0.020 ± 0.002	MS
**DS186**	*Festuca arundinacea* Schreber	Fall	2020	Spalding	University grounds	*C. jacksonii*	0.041 ± 0.003	MS	0.685 ± 0.120	MR
**DS187**	*Zoysia* sp.	Summer	2020	Fulton	Homeowner	*C. monteithiana*	0.654 ± 0.1	LS	0.145 ± 0.014	LR
**DS188**	*Agrostis stolonifera* L.	Fall	2020	Spalding	University grounds	*C. jacksonii*	0.002 ± 0.0004	HS	0.052 ± 0.001	LS
**DS189**	*Cynodon dactylon* L.	Summer	2020	Spalding	Landscape	*C. monteithiana*	>1000	HR	0.217 ± 0.086	MR
**DS190**	*Paspalum vaginatum* Swartz	Summer	2020	Spalding	University grounds	*C. monteithiana*	0.004 ± 0.001	HS	0.071 ± 0.010	LS
**DS191**	*Zoysia* sp.	Summer	2020	Spalding	Landscape	*C. monteithiana*	0.046 ± 0.004	MS	0.109 ± 0.006	LR
**DS192**	*Cynodon dactylon* L.	Summer	2020	Coweta	Homeowner	*C. monteithiana*	0.038 ± 0.003	MS	0.123 ± 0.027	LR
**DS193**	*Zoysia* sp.	Fall	2020	Fulton	Homeowner	*C. monteithiana*	0.006 ± 0.001	HS	0.103 ± 0.007	LR
**DS194**	*Zoysia* sp.	Summer	2020	Upson	Landscape	*C. monteithiana*	0.002 ± 0.002	HS	0.005 ± 0.000	HS
**DS10**	*Zoysia* sp.	Spring	2021	Spalding	University grounds	*C. monteithiana*	0.028 ± 0.005	MS	0.073 ± 0.030	LS
**DS11**	*Cynodon dactylon* L.	Spring	2021	Spalding	University grounds	*C. monteithiana*	0.045 ± 0.02	MS	0.139 ± 0.010	LR
**DS12**	*Cynodon dactylon* L.	Summer	2021	Fulton	Golf Course	*C. monteithiana*	0.050 ± 0.03	MS	0.117 ± 0.004	LR
**DS13**	*Cynodon dactylon* L.	Summer	2021	Lincolnton	Golf Course	*C. monteithiana*	0.003 ± 0.0007	HS	0.061 ± 0.004	LS
**DS14**	*Zoysia* sp.	Summer	2021	Clarke	Homeowner	*C. monteithiana*	0.003 ± 0.0008	HS	0.811 ± 0.232	MR
**DS15**	*Cynodon dactylon* L.	Summer	2021	Fayette	Landscape	*C. monteithiana*	0.025 ± 0.003	MS	0.761 ± 0.097	MR
**DS16**	*Cynodon dactylon* L.	Summer	2021	Fayette	Landscape	*C. monteithiana*	0.008 ± 0.0009	HS	0.041 ± 0.020	MS
**DS17**	*Axonopus fissifolius*	Summer	2021	Fayette	Homeowner	*C. monteithiana*	0.035 ± 0.01	MS	1.400 ± 0.292	HR
**DS18**	*Cynodon dactylon* L.	Summer	2021	Fayette	Landscape	*C. monteithiana*	0.022 ± 0.007	MS	0.068 ± 0.008	LS
**DS19**	*Cynodon dactylon* L.	Summer	2021	Fayette	Landscape	*C. monteithiana*	0.028 ± 0.009	MS	0.086 ± 0.006	LS
**DS20**	*Zoysia* sp.	Summer	2021	Coweta	Homeowner	*C. monteithiana*	0.104 ± 0.007	LS	0.087 ± 0.001	LS
**DS21**	*Cynodon dactylon* L.	Summer	2021	Columbia	Landscape	*C. monteithiana*	0.092 ± 0.04	MS	0.126 ± 0.007	LR
**DS22**	*Cynodon dactylon* L.	Summer	2021	Greene	Landscape	*C. monteithiana*	0.062 ± 0.006	MS	0.177 ± 0.059	LR
**DS23**	*Cynodon dactylon* L.	Summer	2021	Morgan	Landscape	*C. monteithiana*	0.015 ± 0.0086	MS	1.059 ± 0.526	HR
**DS24**	*Cynodon dactylon* L.	Summer	2021	Spalding	Landscape	*C. monteithiana*	0.033 ± 0.0056	MS	0.098 ± 0.020	LS
**DS25**	*Cynodon dactylon* L.	Summer	2021	Rockdale	Landscape	*C. monteithiana*	0.020 ± 0.0016	MS	0.166 ± 0.031	LR
**DS26**	*Cynodon dactylon* L.	Summer	2021	Henry	Landscape	*C. monteithiana*	0.089 ± 0.005	MS	0.836 ± 0.087	MR
**DS27**	*Cynodon dactylon* L.	Summer	2021	Newton	Landscape	*C. monteithiana*	0.392 ± 0.07	LS	1.173 ± 0.297	HR
**DS28**	*Zoysia* sp.	Summer	2021	Harris	Homeowner	*C. monteithiana*	0.002 ± 0.0009	HS	0.325 ± 0.026	MR
**DS29**	*Cynodon dactylon* L.	Summer	2021	Harris	Homeowner	*C. monteithiana*	0.045 ± 0.01	MS	0.722 ± 0.277	MR
**DS195**	*Eremochloa ophiuroides*	Summer	2021	Tift	University grounds	*C. monteithiana*	0.023 ± 0.009	MS	0.698 ± 0.148	MR
**DS196**	*Eremochloa ophiuroides*	Summer	2021	Tift	University grounds	*C. monteithiana*	0.014 ± 0.002	MS	0.058 ± 0.008	LS
**DS197**	*Eremochloa ophiuroides*	Summer	2021	Tift	University grounds	*C. monteithiana*	0.031 ± 0.006	MS	0.123 ± 0.016	LR
**DS198**	*Eremochloa ophiuroides*	Summer	2021	Tift	University grounds	*C. monteithiana*	0.023 ± 0.0008	MS	0.101 ± 0.014	LR
**DS199**	*Eremochloa ophiuroides*	Summer	2021	Tift	Homeowner	*C. monteithiana*	0.006 ± 0.0006	HS	1.101 ± 0.706	HR
**DS200**	*Eremochloa ophiuroides*	Summer	2021	Tift	Homeowner	*C. monteithiana*	0.002 ± 0.0002	HS	0.138 ± 0.011	LR
**DS201**	*Eremochloa ophiuroides*	Summer	2021	Tift	Homeowner	*C. monteithiana*	0.001 ± 0.0002	HS	0.053 ± 0.014	LS
**DS202**	*Digitaria* sp.	Summer	2021	Tift	University grounds	*C. monteithiana*	0.001 ± 0.0004	HS	3.820 ± 0.053	HR
**DS203**	*Digitaria* sp.	Summer	2021	Tift	University grounds	*C. monteithiana*	0.003 ± 0.0004	HS	0.155 ± 0.035	LR
**DS204**	*Digitaria* sp.	Summer	2021	Tift	University grounds	*C. monteithiana*	0.002 ± 0.0002	HS	0.199 ± 0.068	LR
**DS205**	*Digitaria* sp.	Summer	2021	Tift	University grounds	*C. monteithiana*	0.001 ± 0.0005	HS	2.563 ± 0.096	HR
**DS84**	*Stenotaphrum secundatum*	Summer	2022	Lamar	Landscape	*C. monteithiana*	0.025 ± 0.006	MS	0.226 ± 0.150	MR
**DS85**	*Cynodon dactylon* L.	Summer	2022	Hancock	Athletic Field	*C. monteithiana*	0.034 ± 0.002	MS	0.062 ± 0.006	LS
**DS86**	*Cynodon dactylon* L.	Summer	2022	Oconee	Homeowner	*C. monteithiana*	0.035 ± 0.001	MS	0.717 ± 0.047	MR
**DS87**	*Cynodon dactylon* L.	Summer	2022	Taliaferro	Landscape	*C. monteithiana*	0.042 ± 0.001	MS	0.055 ± 0.002	LS
**DS88**	*Cynodon dactylon* L.	Summer	2022	Jasper	Landscape	*C. monteithiana*	0.030 ± 0.002	MS	0.148 ± 0.005	LR
**DS89**	*Cynodon dactylon* L.	Summer	2022	Butts	Landscape	*C. monteithiana*	0.024 ± 0.003	MS	0.136 ± 0.006	LR
**DS90**	*Cynodon dactylon* L.	Summer	2022	Barrow	Landscape	*C. monteithiana*	0.024 ± 0.001	MS	0.073 ± 0.008	LS
**DS91**	*Cynodon dactylon* L.	Summer	2022	Putnam	Landscape	*C. monteithiana*	0.024 ± 0.004	MS	0.042 ± 0.005	MS
**DS92**	*Cynodon dactylon* L.	Summer	2022	Oglethorpe	Landscape	*C. monteithiana*	0.030 ± 0.002	MS	0.079 ± 0.005	LS
**DS93**	*Cynodon dactylon* L.	Summer	2022	Hall	Landscape	*C. monteithiana*	0.050 ± 0.003	MS	0.056 ± 0.005	LS
**DS94**	*Cynodon dactylon* L.	Summer	2022	Union	Landscape	*C. monteithiana*	0.072 ± 0.004	MS	0.444 ± 0.007	MR
**DS95**	*Cynodon dactylon* L.	Summer	2022	White	Landscape	*C. monteithiana*	0.093 ± 0.007	MS	0.041 ± 0.006	MS
**DS96**	*Cynodon dactylon* L.	Summer	2022	Stephens	Landscape	*C. monteithiana*	0.172 ± 0.019	LS	0.093 ± 0.010	LS
**DS97**	*Cynodon dactylon* L.	Summer	2022	Lumpkin	Landscape	*C. monteithiana*	0.095 ± 0.026	MS	0.064 ± 0.013	LS
**DS98**	*Cynodon dactylon* L.	Summer	2022	Clayton	Landscape	*C. monteithiana*	0.058 ± 0.005	MS	0.071 ± 0.004	LS
**DS99**	*Cynodon dactylon* L.	Summer	2022	Rabun	Landscape	*C. monteithiana*	0.069 ± 0.003	MS	1.625 ± 0.110	HR
**DS100**	*Cynodon dactylon* L.	Summer	2022	Franklin	Landscape	*C. monteithiana*	0.038 ± 0.006	MS	0.087 ± 0.007	LS
**DS101**	*Cynodon dactylon* L.	Summer	2022	Forsyth	Landscape	*C. monteithiana*	0.125 ± 0.030	LS	0.129 ± 0.016	LR
**DS102**	*Cynodon dactylon* L.	Summer	2022	Towns	Landscape	*C. monteithiana*	0.065 ± 0.007	MS	0.561 ± 0.093	MR
**DS103**	*Cynodon dactylon* L.	Summer	2022	Banks	Landscape	*C. monteithiana*	0.050 ± 0.0001	MS	0.620 ± 0.076	MR
**DS104**	*Cynodon dactylon* L.	Summer	2022	Madison	Landscape	*C. monteithiana*	0.043 ± 0.005	MS	0.254 ± 0.019	MR
**DS105**	*Zoysia* sp.	Summer	2022	Habersham	Landscape	*C. monteithiana*	0.035 ± 0.0007	MS	0.161 ± 0.009	LR
**DS106**	*Cynodon dactylon* L.	Summer	2022	Wilkes	Golf Course	*C. monteithiana*	0.014 ± 0.0005	MS	0.183 ± 0.059	LR
**DS107**	*Cynodon dactylon* L.	Summer	2022	Jefferson	Athletic Field	*C. monteithiana*	0.048 ± 0.004	MS	0.399 ± 0.109	MR
**DS108**	*Cynodon dactylon* L.	Summer	2022	Warren	Landscape	*C. monteithiana*	0.041 ± 0.002	MS	0.334 ± 0.039	MR

a Common names of host turfgrass are as follows: *Zoysia* sp. = Zoysiagrass; *Paspalum vaginatum* Swartz = Seashore Paspalum; *Agrostis stolonifera* L. = Creeping Bentgrass; *Cynodon dactylon* L. = Bermudagrass; *Digitaria* sp. = Crabgrass; *Festuca arundinacea* Schreber = Tall Fescue; *Axonopus fissifolius* = Carpetgrass; *Eremochloa ophiuroides* = Centipedegrass; *Stenotaphrum secundatum* = St. Augustinegrass. Color coding identifies the year of collection.

b EC_50_ threshold for thiophanate-methyl is as follows: highly sensitive (HS): 0.001 to 0.01 μg/mL; moderate sensitive (MS): >0.01 to 0.1 μg/mL; low sensitive (LS): >0.1 to 1.0 μg/mL; low resistant (LR): >1.0 to 5.0 μg/mL; moderate resistant (MR): >5.0 to 100.0 μg/mL; and highly resistant (HR): >100.0 μg/mL.

c EC_50_ threshold for propiconazole is as follows: HS: <0.01 μg/mL; MS: 0.01 to 0.05 μg/mL; LS: >0.05 to 0.1 μg/mL; LR: >0.1 to 0.2 μg/mL; MR: >0.2 to 1.0 μg/mL; and HR: >1.0 μg/mL.

### 2.2 Bio- and synthetic fungicide preparations

Technical-grade fungicides were used for *in vitro* fungicide sensitivity. Thiophanate-methyl (98.0% a.i.), and propiconazole (98.5% a.i.) were purchased from Sigma-Aldrich (Sigma-Aldrich, Inc. St. Louis, MO). Each fungicide was dissolved in acetone (0.1% v/v) to prepare stock solutions (1000 μg/mL) and stored at 4°C in the dark to preserve fungicide activity. 100 mg of technical-grade fungicides were first dissolved in 1 ml of acetone and diluted in 99 ml solvent to make 1000 μg/mL stock solution. Further, three bio-fungicides including *B. subtilis* QST713 (Rhapsody™), *B. amyloliquefaciens* F727 (Stargus™), and *R. sachalinensis* extr. (Regalia™) along with six synthetic fungicides including azoxystrobin (Heritage™), fludioxonil (Medallion™), fluxapyroxad (Xzemplar™), penthiopyrad (Velista™), propiconazole (Banner x™), and boscalid (Emerald™) were tested. These fungicides were used against *C. monteithiana* for *in vitro* tests and for their efficacy against dollar spot in the growth chamber and field experiments. Fungicides were prepared as per manufacturer instructions and applied on the same day of preparation (detailed information on formulations, rates, and manufacturers can be found in **Table S1**).

### 2.3 *In vitro* fungicide sensitivity to propiconazole and thiophanate-methyl

Fungicide sensitivity assays were performed on the 79 *Clarireedia* spp. isolates and followed the procedures outlined in Jo et al. (2006). *Clarireedia* spp. isolates were cultured on PDA at 25°C in the dark for 4 days. A 4 mm diameter agar plug taken from the edge of an actively growing colony was placed at the center of the PDA plate amended with 0, 0.001, 0.01, 0.1, and 1 µg/ml propiconazole or 0, 0.001, 0.01, 0.1, 1, 10, 100, and 1000 µg/ml thiophanate-methyl. Fungicide solutions were added to autoclaved PDA cooled to 50°C. Agar plates amended with the same concentration of acetone (0.1% v/v) were used as a control. For each concentration, triplicate plates were utilized and the plates were incubated at 25°C in the dark. After 48 hours, two perpendicular colony diameters were measured per plate, and the mean was used to compute the colony diameter for each concentration. Mycelial radial growth was measured and percent inhibition was calculated as: (colony diameter of control - colony diameter of treatment)/(colony diameter of control - mycelial disk diameter) × 100. EC_50_ (the concentration inhibiting mycelial growth by 50%) values for each isolate was calculated in SigmaPlot (Systat Software Inc., San Jose, CA) by solving linear regression equations of the probit-transformed percent growth data versus the base-10 logarithm of each fungicide concentration. The fungicide-resistant classification for thiophanate-methyl and propiconazole in this study was according to Hu et al. (2018) and Jo et al. (2006), respectively, with some modifications. For thiophanate-methyl, isolates with an EC_50_ value of 0.001 to 0.01 μg/mL were considered highly sensitive (HS), >0.01 to 0.1 μg/mL were moderate sensitive (MS), >0.1 to 1.0 μg/mL were low sensitive (LS), >1.0 to 5.0 μg/mL were low resistant (LR), >5.0 to 100.0 μg/mL were moderate resistant (MR), and >100.0 μg/mL were highly resistant (HR). For propiconazole, isolates with an EC_50_ value <0.01 μg/mL were considered HS, 0.01 to 0.05 μg/mL were MS, >0.05 to 0.1 μg/mL were LS, >0.1 to 0.2 μg/mL were LR, >0.2 to 1.0 μg/mL were MR, and >1.0 μg/mL were HR.

### 2.4 *In vitro* bio- and synthetic fungicide efficacy assays

Two-fold *in vitro* experiments were conducted. First, the efficacy of the three bio-fungicides was tested *in vitro* against *C. monteithiana* isolate DS8 along with six synthetic fungicides at their label rates (**Tables S1, S2**). The objective was to select the best synthetic fungicide. The experiment was set up as a completely randomized design with five replications where each petri dish (100 × 15 mm) served as an individual replicate. The *in vitro* experiment was conducted using the ‘poisoned food technique’ as described by Grover and Moore (1962). Since fungal mycelial growth outcompeted the Petri- plate diameter prior to four days after incubation when the agar plug was placed in the center, the fungal plug was transferred from a pure culture to one periphery of the fungicide-amended PDA plate instead. The longest mycelial radial growth was measured after four days of incubation at 25°C in a 12-hour photoperiod and percent growth inhibition for each fungicide treatment in reference to the non-fungicide amended control plate was calculated using the formula used in the sensitivity assays. The experiment was repeated. Next, ten different combinations of bio- and synthetic fungicides comprising of *B. subtilis* QST713, *R. sachalinensis* extr*.*, and propiconazole at varying tank mix ratios of their label rates [25:75 (1 part synthetic fungicide at label rate and 3 parts biofungicide at label rate), 50:50, and 75:25] along with non-fungicide amended control were assayed to identify the best treatment combination that could further be tested in the growth chamber and field experiments (**Table S2**). Experimental setup, data collection techniques, and parameters followed as described previously in the sensitivity assays.

### 2.5 Growth chamber bio- and synthetic fungicide efficacy experiments

The efficacy of biofungicide *B. subtilis* QST713 was tested together with propiconazole either alone or in tank-mix combinations in a growth chamber at the University of Georgia, Griffin Campus, Griffin, GA in 2022. Turf plugs (7.6 cm × 7.6 cm)) were collected from a bermudagrass cv ‘TifTuf’ [Bermuda grass cv. ‘TifTuf’ is an interspecific triploid hybrid of *C. transvaalensis* and *C. dactylon*, was co-released by the University of Georgia and the U.S. Department of Agriculture–Agricultural Research Service in 2014 and is susceptible to dollar spot (Hanna and Schwartz, 2016)] field and were grown for two months in a Sungro professional growing mix (Sun Gro Horticulture, MA, USA) in the greenhouse. The grass was trimmed to 5 cm and fertilized with 0.7 gm/L water of Miracle-Gro^®^ Water Soluble All Purpose Plant Food (The Scotts Company LLC, USA) every week and inspected to maintain the plant materials free of disease before inoculation. Nitrogen fertilization was cut off two weeks before the start of the experiment and pots were flushed with water to favor dollar spot infection. The experiment was laid as a repeated measure randomized complete block (RCB) design with four replicated pots. The turf was inoculated by hand-dispersal of four grains infested with the *C. monteithiana* isolate DS8 into the foliar canopy and maintained under high humidity conditions for 48 hours in the greenhouse. Inoculated pots were then incubated in a growth chamber (25 and 16°C day/night with a 12-hour photoperiod) for seven days to initiate dollar spot infection, after which the spray program started (also termed as 0 day after the start of the experiment), and pots were kept in the growth chamber until 42 days. The term ‘spray program’ is used in our study, which is equivalent to treatment, because we applied more than one fungicides (bio and synthetic) at different time intervals in the individual treatment. The seven spray programs included: 1) non-treated control with no fungicide application (T1), 2) *B. subtilis* QST713 applied every 7 days (T2), 3) *B. subtilis* QST713 applied every 14 days (T3), 4) propiconazole applied every 28 days (T4), 5) tank mix of 75% *B. subtilis* QST713 + 25% propiconazole applied every 28 days (T5), 6) 75% *B. subtilis* QST713 + 25% propiconazole tank mix in rotation with 100% *B. subtilis* QST713 applied every 14 days (T6), and 7) 100% *B. subtilis* QST713 in rotation with 75% *B. subtilis* QST713 + 25% propiconazole tank mix applied every 14 days (T7) (**Table S3**). Visual estimates of dollar spot severity was noted at weekly intervals on each pot using a modified Horsfall-Barratt scale of 1-11 (Horsfall and Barrett, 1945) starting from 0 to 42 days for a total of seven time points. The experiment was repeated. The disease severity data was transformed to a percent scale using ARM statistical software (GDM Solutions, Inc., Brookings, SD). Percent of disease severity and area under the disease progress curve (AUDPC) were subjected to analysis of variance using R statistical software (R Core Team, 2021) and means were separated using Tukey’s HSD test (*P* = 0.05).

### 2.6 Field bio- and synthetic fungicide efficacy experiments

The bio- and synthetic fungicide efficacy experiments were conducted on two separate plots of a 3-year bermudagrass cv ‘TifTuf’ grown on clay loam soil (pH = 5.8) for two seasons (summer and fall) during 2022 at the UGA, Griffin Campus in Griffin, GA. Turfgrass was maintained based on the best-recommended management practices for golf course fairways in GA (GCSAA, 2018). Turf was irrigated every evening to maintain high humidity and mowed to 1.6 cm once a week. Nitrogen fertilization was not applied to the experimental plots during the study. The experiment was arranged as an RCB design in 0.91 m × 0.91 m plots with four replications. Because the summer season (Jun to Jul 2022) was more favorable for natural dollar spot infection in the experimental plots, artificial inoculations with *C. monteithiana* isolate DS8 was only applied during the fall season trial (Sep to Nov 2022) by uniformly spreading 20 g of dollar spot-infected grain inoculum per experimental plot a week prior to the start of fungicide spray programs. The inoculation was repeated after five days to ensure that abundant dollar spot epidemics would be developed. The seven spray programs that were already tested in growth chambers, including a non-treated control, were evaluated from Jun 09 to Jul 21, 2022 (summer season) and Sep 26 to Nov 07, 2022 (fall season) (**Table S4**). Applications started one week after the first inoculation at the rate of 81.5 mL of water per sq. m. with a hand-held CO_2_-pressured boom sprayer at 30 psi using an XR TeeJet 8004VS nozzle. Dollar spot severity was visually assessed every week on each experimental plot using a modified Horsfall-Barratt rating scale (1-11) starting from 0 day (start of the spray program) until 42 days (end of the spray program) for a total of seven time points (Horsfall & Barratt, 1945). Dollar spot severity was transformed to percent disease severity using ARM statistical software, as described above. Turf quality was also rated every week using the national turfgrass evaluation program (NTEP) 1-9 ratings, where 1 is poor/dead, 7 is minimally acceptable, and 9 is excellent (Lee et al., 2011). Data on percent disease severity, turf quality, and AUDPC were subjected to analysis of variance using the R statistical package, and means were separated using Tukey’s HSD test (*P* = 0.05).

## 3 Results

### 3.1 *In vitro* sensitivity of *Clarireedia* spp to thiophanate-methyl and propiconazole

**Figure 1:**
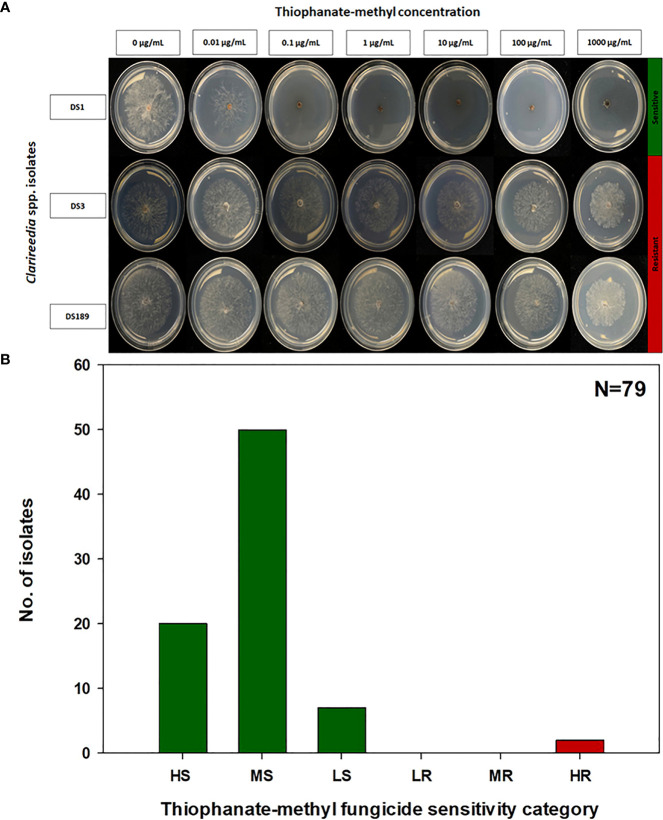
Thiophanate-methyl fungicide sensitivity assays **(A)** and distribution **(B)** of sensitive and resistant isolates to thiophanate-methyl for 79 *Clarireedia* spp. isolates collected between 2019 to 2022 in the state of Georgia. HS: highly sensitive (0.001 to 0.01 μg/mL); MS: moderate sensitive (>0.01 to 0.1 μg/mL); LS: low sensitive (>0.1 to 1.0 μg/mL); LR: low resistant (>1.0 to 5.0 μg/mL); MR: moderate resistant (>5.0 to 100.0 μg/mL); and HR: highly resistant (>100.0 μg/mL).

**Figure 2:**
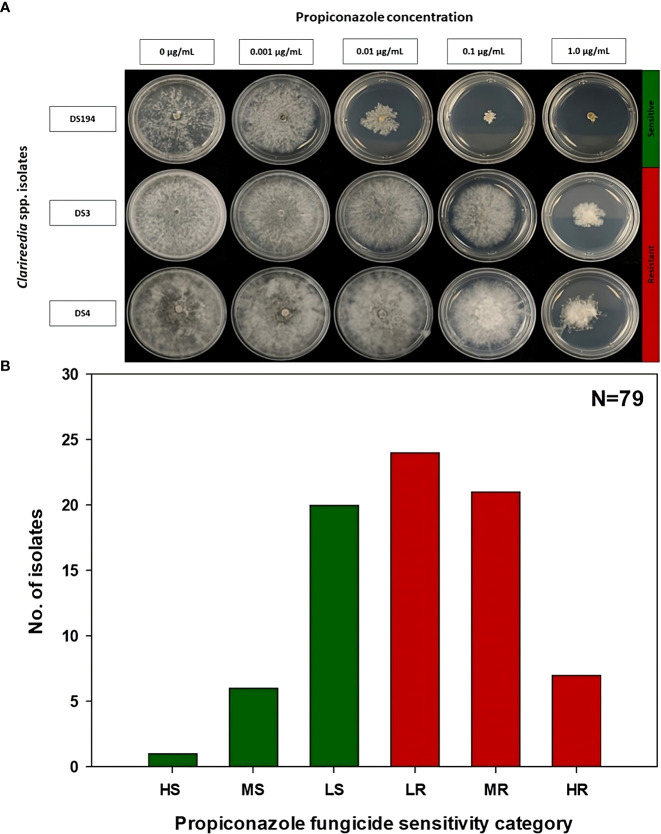
Propiconazole fungicide sensitivity assays **(A)** and distribution **(B)** of sensitive and resistant isolates to propiconazole for 79 *Clarireedia* spp. isolates collected between 2019 to 2022 in the state of Georgia. HS: highly sensitive (<0.01 μg/mL); MS: moderate sensitive (0.01 to 0.05 μg/mL); LS: low sensitive (>0.05 to 0.1 μg/mL); LR, low resistant (>0.1 to 0.2 μg/mL); MR, moderate resistant (>0.2 to 1.0 μg/mL); and HR, highly resistant (>1.0 μg/mL).

The EC_50_ of the 79 *Clarireedia* spp. isolates for thiophanate-methyl ranged from 0.001 to >1000.0 μg/mL (**Table 1**). Seventy-seven isolates (97.5%) were sensitive [20 HS (25%), 50 MS (63.3%), and 7 LS (8.9%)] to thiophanate-methyl and their mean EC_50_ value was 0.052 ± 0.092 μg/mL. No LR and MR isolates were identified; however, only two isolates (2.5%) were highly resistant (HR) to thiophanate-methyl among which one was *C. jacksonii* and the other was *C. monteithiana* sampled from Spalding county in 2019 and 2020, respectively (**Figures 1A, B**, **Table 1**). However, no resistant isolates to thiophanate-methyl were found in 2021 and 2022. The EC_50_ of the 79 isolates for propiconazole ranged from 0.005 to 3.820 μg/mL (**Table 1**). Twenty-seven isolates (34.2%) were sensitive [1 HS (1.3%), 6 MS (7.6%), and 20 LS (25.3%)] while 52 isolates (65.8%) were resistant [24 LR (30.3%), 21 MR (26.6%), and 7 HR (8.9%)] to propiconazole (**Figures 2A, B** and **Table 1**). Two, 14, 22, and 14 *Clarireedia* isolates were found to be resistant each year from 2019 to 2022, respectively. At the species level, out of four *C. jacksonii* isolates, one was resistant and the remaining three were sensitive to thiophanate-methyl. Similarly, two of the *C. jacksonii* isolates were sensitive and the other two were resistant to propiconazole. Among 75 C*. monteithiana* isolates, only one isolate was resistant to thiophanate-methyl whereas 25 were sensitive and 50 were resistant to propiconazole. Overall, 27 isolates (25 C*. monteithiana* and 2 C*. jacksonii*) were sensitive and two isolates (1 C*. monteithiana* and 1 C*. jacksonii*) were resistant to both fungicides propiconazole and thiophanate-methyl (**Table 1**).

### 3.2 Bio- and synthetic fungicide efficacy *in vitro*

**Figure 3:**
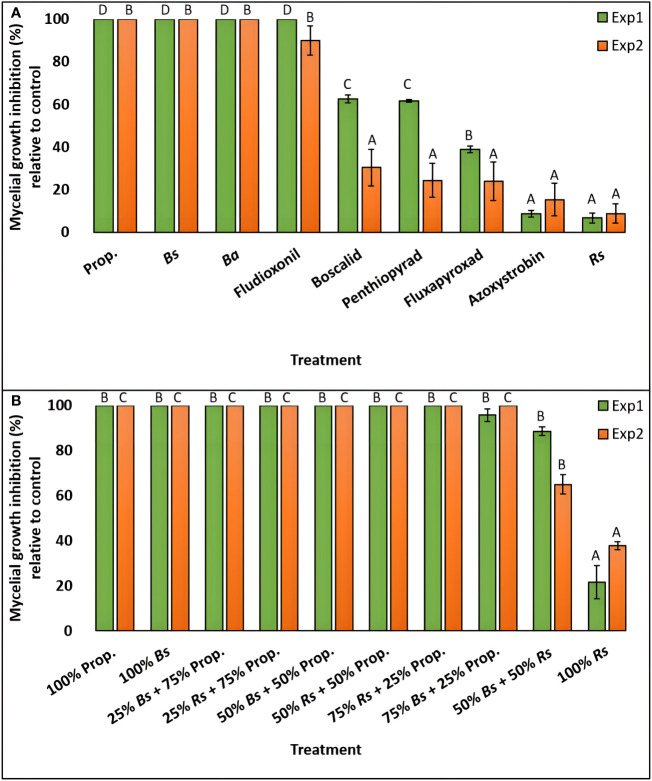
Percent mycelial growth inhibition of *C. monteithiana* grown in three bio- and six synthetic fungicides **(A)**, and ten different combinations of two bio- and one synthetic fungicide-amended potato dextrose agar (PDA) plates at different proportions **(B)** compared to the untreated control on *in vitro* assays in the first (green bar) and second (orange bar) experiments. Mean disease severity with the same letters in the bar chart for an individual experiment are not significantly different according to Tukey’s test (*P* = 0.05) based on separate analysis for experiment 1 (Exp1) and experiment 2 (Exp2). Prop., Propiconazole; *Bs*, *Bacillus subtilis* QST713; *Ba*, *B. amyloliquefaciens* F727; *Rs*, *Reynoutria sachalinensis* extr.

The effect of nine fungicide treatments on *C. monteithiana* (isolate DS8) mycelial growth inhibition across two experiments was performed. Since the ‘experiment’ and ‘treatment × experiment’ effects were significant (*P*<0.05) on mycelial growth inhibition, the two experiments were analyzed separately (**Table S5**). In the first experiment, biofungicides *B. subtilis* QST713 and *B. amyloliquefaciens* F727 and fungicides propiconazole and fludioxonil entirely suppressed the growth of *C. monteithiana* (**Figures 3A** and **S2, Table S2**). Biofungicide *R. sachalinensis* extr. and fungicide azoxystrobin were the least effective of all treatments depicting the lowest mycelial growth inhibition (6.7 and 8.8%, respectively). An intermediate result was obtained for fungicides fluxapyroxad, penthiopyrad, and boscalid which reduced mycelial growth by 39.0, 61.6, and 62.6%, respectively, compared to the control. A similar pattern was observed in the second experiment with *B. subtilis* QST713, *B. amyloliquefaciens* F727, propiconazole, and fludioxonil significantly reducing pathogen growth by 90-100% compared to the control treatment (**Figures 3A** and **S2, Table S2**). All other fungicides reduced mycelial growth by 9-31% and were significantly different (*P*>0.05) from this first group.

To evaluate if tank mixes of biofungicides and synthetic fungicides can provide control against dollar spot, the efficacy of biofungicides *B. subtilis* QST713 and *R. sachalinensis* extr. and synthetic fungicide propiconazole were tested *in vitro* in two independent experiments in different combinations of 1:3, 1:1, and 3:1 and compared with their individual-amended plate and a non-treated control plate. The effect of ten fungicide treatments on mycelial growth inhibition across two experiments was performed. Since there was a significant ‘treatment × experiment’ effect (*P*<0.05) on mycelial growth inhibition, the two experiments were analyzed separately (**Table S5**). In the first experiment, except for the 100% *R. sachalinensis* extr.-amended plate, which demonstrated 22% growth reduction, all other treatments, including 100% propiconazole and 100% *B. subtilis* QST713-amended plate, were significantly similar to each other (*P*>0.05) and were found effective in reducing the fungal growth by 89-100% compared to the control (**Figures 3B** and **S3, Table S2**). The second experiment yielded a similar pattern except that plates amended with 100% *R. sachalinensis* extr. and 50% *B. subtilis* QST713 + 50% *R. sachalinensis* extr. were statistically different from each other and to the rest of the other treatments (*P*<0.05) and reduced mycelial growth by 37.9 and 65.0%, respectively, compared to the control (**Figures 3B** and **S3, Table S2**). All other eight treatments were statistically similar to each other (*P*>0.05) and completely inhibited the mycelial growth of *C. monteithiana* isolate DS8.

### 3.3 Bio- and synthetic fungicide efficacy in growth chamber experiments

The effect of seven spray programs over seven different time points on disease severity and AUDPC was assessed across two experiments in the growth chamber. The effect of the ‘experiment’ was statistically non-significant (*P*>0.05) for disease severity, while a significant effect (*P*<0.05) was observed for the AUDPC (**Table S6**). Therefore, the data from the two experiments were subjected to combined analysis for disease severity and separate analysis for the AUDPC.

#### 3.3.1 Disease severity

**Figure 4:**
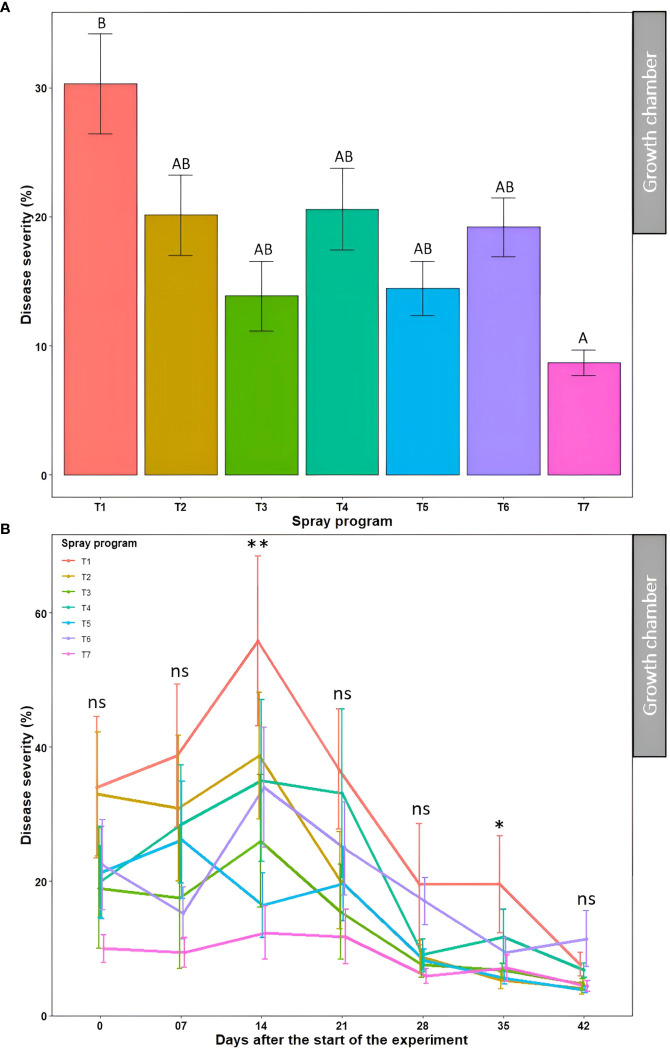
Dollar spot average disease severity (%) for seven spray programs (T1-T7) **(A)** and the interaction of the seven spray programs and seven different time points (0-42 days) **(B)** in the growth chamber across two experiments. Mean disease severity with the same letters in the bar chart in panel A are not significantly different according to Tukey’s test (*P* = 0.05). Tukey’s test showed a significant difference in mean disease severity in panel B across seven spray programs at 14 and 35 days (*P*<0.05). T1: non-treated control; T2: *B. subtilis* QST713 applied every 7 days; T3: *B. subtilis* QST713 applied every 14 days; T4: propiconazole applied every 28 days; T5: tank mix of 75% *B. subtilis* QST713 + 25% propiconazole applied every 28 days; T6: 75% *B. subtilis* QST713 + 25% propiconazole tank mix in rotation with 100% *B. subtilis* QST713 applied every 14 days; and T7: 100% *B. subtilis* QST713 in rotation with 75% *B. subtilis* QST713 + 25% propiconazole tank mix applied every 14 days. *significant at P<0.05, **significant at P<0.01, and ns non-significant at P<0.05.

The average percent disease severity for spray program T7 (100% *B. subtilis* QST713 in rotation with 75% *B. subtilis* QST713 + 25% propiconazole tank mix applied every 14 days) across seven time points was the lowest of all (8.7%) and significantly different (*P*<0.05) from the non-treated control pot. Inversely, five other spray programs (13.9-20.6%) did not differ significantly (*P*>0.05) among each other and from the control plot (**Figure 4A**, **Table S3**). A significantly higher percent disease severity (22.8-31.2%) was observed from 0-21 days across seven spray programs which significantly differed (*P*<0.05) from time points 28, 35, and 42 days noting lower percent severity (10.9, 9.3, and 6.1%, respectively) (**Table S3**). The interaction effect of ‘spray program × time’ for percent disease severity was found non-significant (*P*>0.05) (**Table S6**). We observed a decline in disease severity after 14 days probably due to the lower relative humidity (60%) registered in the growth chamber experiments compared to the optimal (>90%) for dollar spot development (**Figure 4B**). Still, significant differences (*P*<0.05) in percent disease severities were observed among seven spray programs at 14 and 35 days with the non-treated control pot recording the lowest disease severity of all. Among all 49 data points, comprising seven spray programs and seven time points, the lowest disease severity was noted in the treatment sprayed with a tank mix of 75% *B. subtilis* QST713 + 25% propiconazole applied every 28 days at 42 days after the start of the experiment (3.8%). Taken together, all five spray programs comprising biofungicide were as effective as applying synthetic fungicide alone and reduced dollar spot severity to a maximum of 71% compared to the non-treated control (**Table S3**).

#### 3.3.2 Area under the disease progress curve

**Figure 5:**
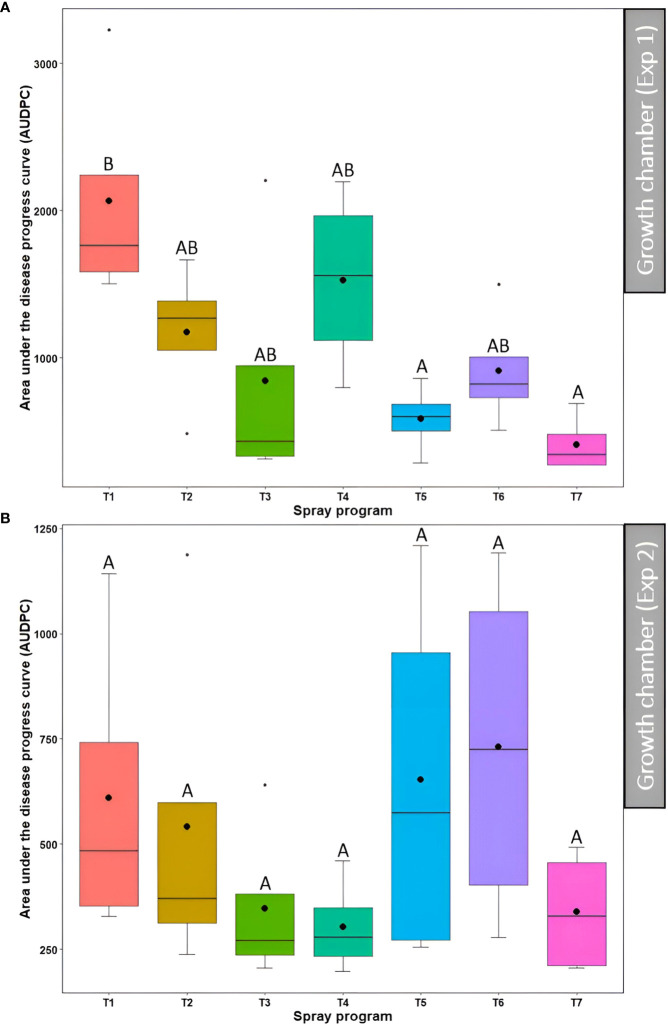
Area under the disease progress curve (AUDPC) of dollar spot resulting from the application of seven different spray programs (T1-T7) in the growth chamber during the first **(A)** and second **(B)** experiments. Mean AUDPC with the same letters in the box plot are not significantly different according to Tukey’s test (*P*<0.05). T1: non-treated control; T2: *B. subtilis* QST713 applied every 7 days; T3: *B. subtilis* QST713 applied every 14 days; T4: propiconazole applied every 28 days; T5: tank mix of 75% *B. subtilis* QST713 + 25% propiconazole applied every 28 days; T6: 75% *B. subtilis* QST713 + 25% propiconazole tank mix in rotation with 100% *B. subtilis* QST713 applied every 14 days; and T7: 100% *B. subtilis* QST713 in rotation with 75% *B. subtilis* QST713 + 25% propiconazole tank mix applied every 14 days.

In the first experiment, a significant difference (*P*<0.05) in the AUDPC was observed across seven treatment programs among which non-treated control had the higher AUDPC (2064.4) whereas lower AUDPC was observed for the spray programs including a tank mix of 75% *B. subtilis* QST713 + 25% propiconazole applied every 28 days (586.4) and 100% *B. subtilis* QST713 in rotation with a tank mix of 75% *B. subtilis* QST713 + 25% propiconazole applied every 14 days (410.0) (**Figure 5A**, **Table S3**). The AUDPC for all other spray programs fell in the intermediate range (844.1-1526.4) and noted no statistical differentiation (*P*>0.05) with any of these two extremities. Nonetheless, the effect of the spray program on the AUDPC was found non-significant (*P*>0.05) in the second experiment with six spray programs (374.1-914.8) holding no significant differences from the non-treated control pots (1337.0) (**Figure 5B**, **Table S3**).

### 3.4 Bio- and synthetic fungicide efficacy in field experiments

The effect of seven spray programs over seven different time points on disease severity, turf quality, and AUDPC was assessed across two seasons in the field. The effects of ‘season’, ‘season × spray program’, and ‘season × spray program × time’ were statistically non-significant (*P*>0.05) for disease severity, turf quality, and AUDPC (**Table S7**). Therefore, the data from the summer and fall seasons were subjected to combined analysis.

#### 3.4.1 Disease severity

**Figure 6:**
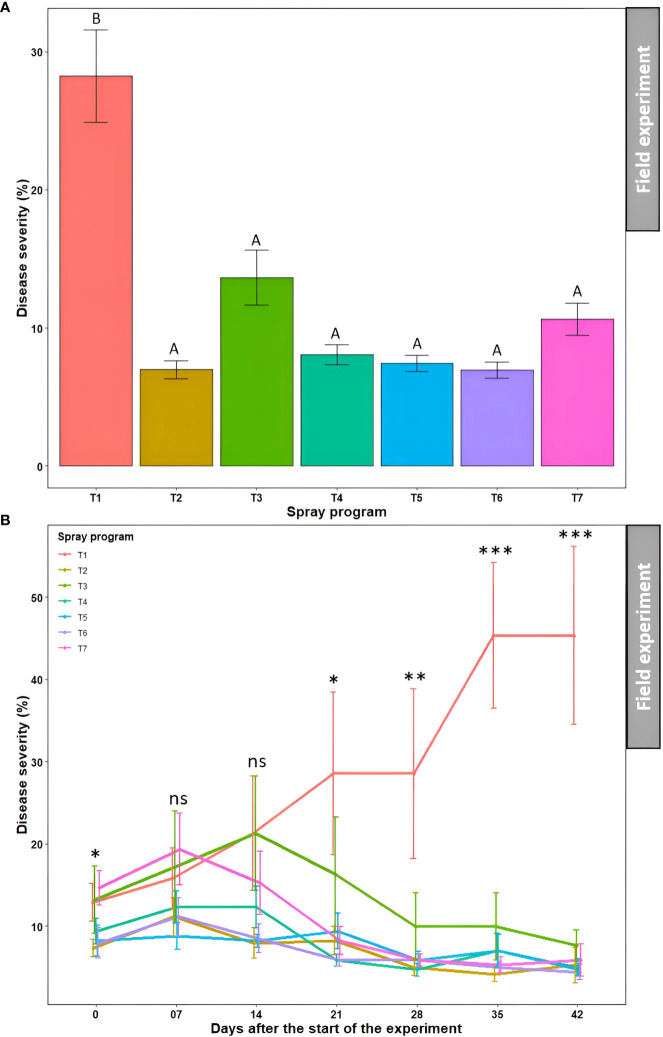
Dollar spot average disease severity (%) for seven spray programs (T1-T7) **(A)** and the interaction of the seven spray programs and seven different time points (0-42 days) **(B)** in the field experiments across both summer and fall seasons. Mean disease severity with the same letters in the bar chart in panel A are not significantly different according to Tukey’s test (*P* = 0.05). Tukey’s test showed a significant difference in mean disease severity in panel B across seven spray programs at 0, 21, 28, 35, and 42 days (*P*<0.05). T1: non-treated control; T2: *B. subtilis* QST713 applied every 7 days; T3: *B. subtilis* QST713 applied every 14 days; T4: propiconazole applied every 28 days; T5: tank mix of 75% *B. subtilis* QST713 + 25% propiconazole applied every 28 days; T6: 75% *B. subtilis* QST713 + 25% propiconazole tank mix in rotation with 100% *B. subtilis* QST713 applied every 14 days; and T7: 100% *B. subtilis* QST713 in rotation with 75% *B. subtilis* QST713 + 25% propiconazole tank mix applied every 14 days. *significant at P<0.05, **significant at P<0.01, ***significant at P<0.001, and ns non-significant at P<0.05.

Overall, all six spray programs were found effective in controlling dollar spot by reducing disease severity by 52-75% in the field when compared to the non-treated control (**Table S4**). The average percent disease severity across seven time points for all six spray programs (7.0-13.7%) were significantly different from the non-treated control (28.2%) (*P*<0.05); however, they were not significantly different from each other (*P*>0.05) (**Figures 6A** and **S4, Table S4**). No significant difference (*P*>0.05) in average percent disease severity was observed across seven spray programs for seven time points (9.4-13.7%) (**Table S4**). The two-way ANOVA revealed a significant ‘spray program × time’ interaction (*P*>0.05) where the percent disease severity continued to escalate in the non-treated control plots with no significant difference (*P*>0.05) with the six other spray programs until 14 days after the start of the experiment (**Figure 6B**). However, the average disease severities started to decline for the six spray programs and were significantly different from the non-treated control starting 21 days (*P*>0.05). The disease severity reached a plateau at 35 days with the significantly (*P*<0.05) highest severity recorded as 45.3% for the non-treated control plot. At 42 days, all six spray programs resulted in significantly (*P*<0.05) lower disease severities ranging from 4.4 to 7.6% compared to the non-treated control (**Figure 6B**). Among all 49 data points, comprising seven spray programs and seven time points, the lowest disease severity was noted in the plot sprayed with *B. subtilis* QST713 every 7 days interval at 35 days after the start of the experiment (4.1%).

#### 3.4.2 Turf quality

**Figure 7:**
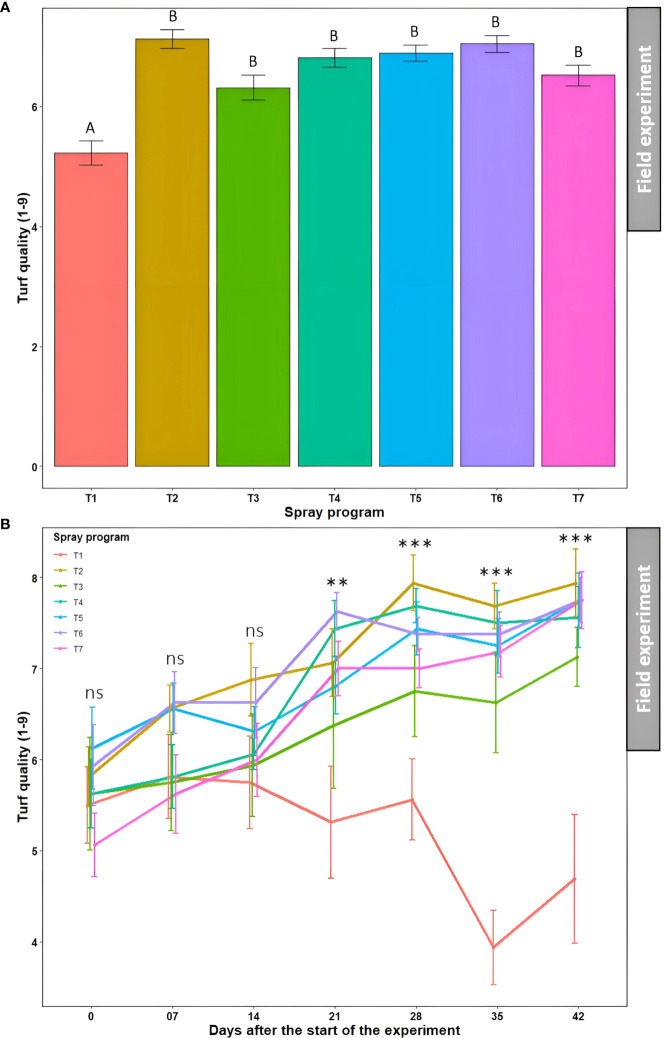
Turf quality (1-9) resulting from the application of seven spray programs (T1-T7) **(A)** and the interaction of seven spray programs and seven different time points (0-42 days) **(B)** in the field experiments across both summer and fall seasons. Mean turf quality with the same letters in the bar chart in Panel A are not significantly different according to Tukey’s test (*P* = 0.05). Tukey’s test showed a significant difference in mean disease severity in Panel B across seven programs at 21, 28, 35, and 42 days (*P*<0.05). T1: non-treated control; T2: *B. subtilis* QST713 applied every 7 days; T3: *B. subtilis* QST713 applied every 14 days; T4: propiconazole applied every 28 days; T5: tank mix of 75% *B. subtilis* QST713 + 25% propiconazole applied every 28 days; T6: 75% *B. subtilis* QST713 + 25% propiconazole tank mix in rotation with 100% *B. subtilis* QST713 applied every 14 days; and T7: 100% *B. subtilis* QST713 in rotation with 75% *B. subtilis* QST713 + 25% propiconazole tank mix applied every 14 days. *significant at P<0.05, **significant at P<0.01, and ns non-significant at P<0.05.

Significantly lower average turf quality across seven time points was noted in the non-treated control plots (5.2) which statistically (*P*<0.05) differentiated from the rest of the six treated plots (6.3-7.1) (**Figures 7A** and **S4, Table S4**). Nonetheless, these six treatments were statistically similar to each other (*P*>0.05). Average turf quality was significantly lower (*P*<0.05) at 0 day after the start of the experiment (5.7) when averaged across the seven spray programs which kept improving over time attaining the statistically highest quality at 42 days (7.2) (**Table S4**). The two-way ANOVA revealed a significant ‘spray program × time’ interaction (*P*>0.05) and portrayed a tendency for the continued deterioration of turf quality on non-treated control plots over the 42-day experiment period (**Figure 7B**). Inversely, turf quality continually improved for all six treated plots. There were no significant differences (*P*>0.05) in turf quality across the seven spray programs until 14 days, after which the effect of the six spray programs were conspicuous compared to the non-treated control. At 42 days, significantly lower (*P*<0.05) average turf quality was observed in the non-treated plots (4.7) compared to the treated plots that yielded significantly higher turf quality (7.1 to 7.9) showcasing the comparable efficacy of biofungicide to synthetic fungicide (**Figure 7B**).

#### 3.4.3 Area under the disease progress curve

**Figure 8:**
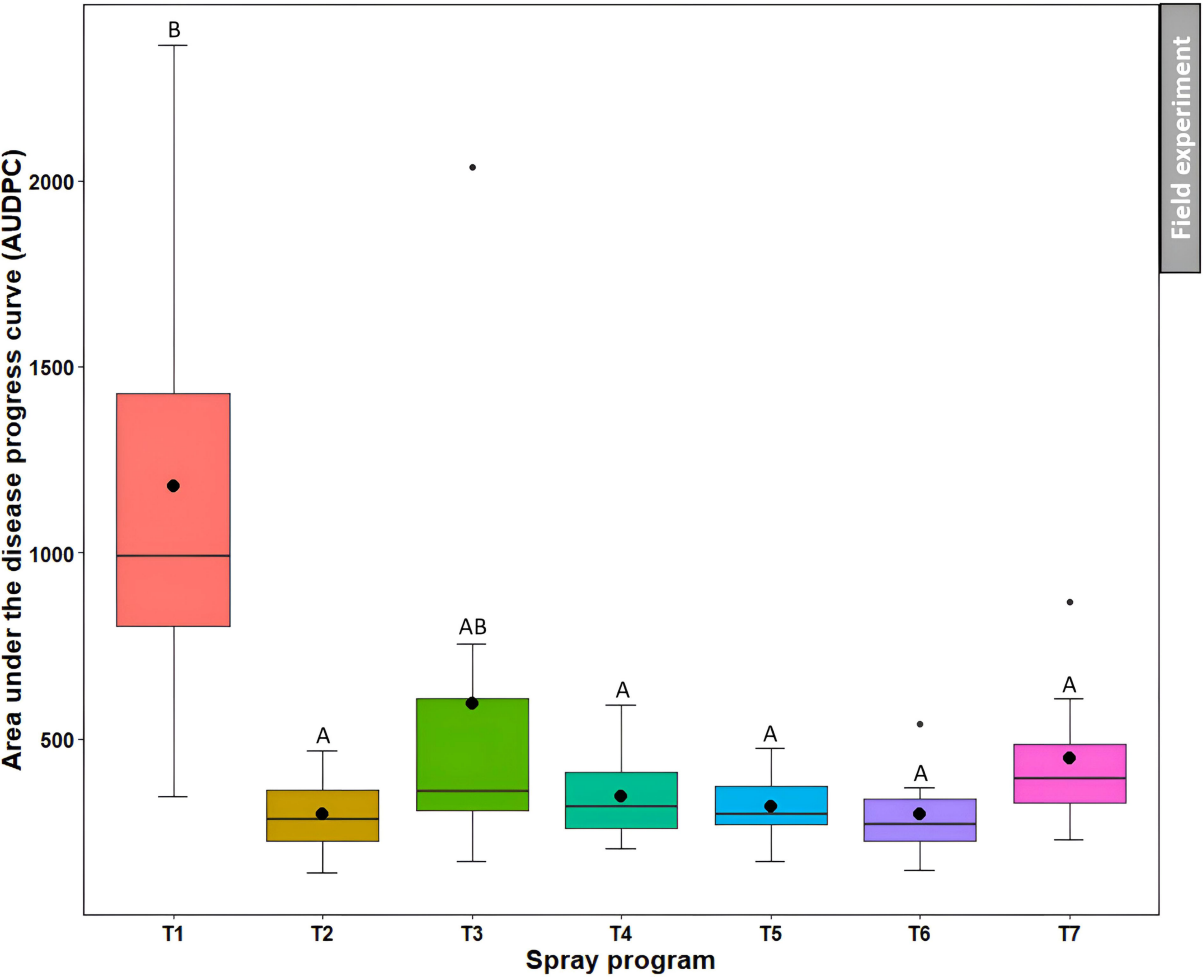
Area under the disease progress curve (AUDPC) of dollar spot resulting from the application of seven different spray programs (T1-T7) in the field experiments across both summer and fall seasons. Mean AUDPC with the same letters in the box plot are not significantly different according to Tukey’s test (*P*<0.05).T1: non-treated control; T2: *B. subtilis* QST713 applied every 7 days; T3: *B. subtilis* QST713 applied every 14 days; T4: propiconazole applied every 28 days; T5: tank mix of 75% *B. subtilis* QST713 + 25% propiconazole applied every 28 days; T6: 75% *B. subtilis* QST713 + 25% propiconazole tank mix in rotation with 100% *B. subtilis* QST713 applied every 14 days; and T7: 100% *B. subtilis* QST713 in rotation with 75% *B. subtilis* QST713 + 25% propiconazole tank mix applied every 14 days.

Non-treated control plots noted the higher AUDPC (1179.8) which was not statistically different (*P*>0.05) from the *B. subtilis* QST713 applied every 14 days (595.6) (**Figure 8**, **Table S4**). However, the other five spray programs resulted in significantly (*P*<0.05) lower AUDPC (range from 297.1 to 448.9) compared to the non-treated control, demonstrating their effectiveness in controlling dollar spot.

## 4 Discussion

With the expansion in the sports turf market witnessed in recent years along with escalating demand of public and commercial urban landscapes, effective and timely management of dollar spot is of utmost importance to maintain the disease-free turfgrass. Dollar spot is primarily managed by fungicides. Resistance to fungicides can result in reduced efficacy, shorter control intervals, reduced turf density, and even complete loss of disease control (Clarke et al., 2020). Resistance to DMI and MBC fungicides has been reported for *C. jacksonii* from the northeast US (Clarke et al., 2020; Koch et al., 2021), however information is limited on sensitivity to these fungicides in Georgia.

Our study revealed 64.8% of the *Clarireedia* spp. isolates resistance to propiconazole, but only 2.5% to thiophanate-methyl. In other US states, 85-96% and 41% of the isolates exhibited resistance to propiconazole and thiophanate-methyl, respectively (Jo et al., 2006; Putman et al., 2010; Stephens and Kaminski, 2019). In the present study, we observed only two isolates out of 79 that exhibited a high level (>1000 μg/mL) of resistance to thiophanate-methyl. This is the first report of thiophanate-methyl sensitivity to *Clarireedia* spp. in Georgia. Hu et al. (2018) found that the EC_50_ of 44 *Clarireedia* spp. isolates collected from seashore paspalum fairways in China ranged from 0.0257 to >1000 μg/mL, and among them, 22 isolates were highly resistant, which is much higher frequency than observed in our study. Baseline sensitivities of *Clarireedia* spp. for propiconazole between 1999-2000 in Georgia ranged from 0.0006 to 0.0102 μg/mL with a mean of 0.0049 μg/mL for propiconazole unexposed (n = 59) and 0.005 to 0.057 μg/mL with a mean of 0.0283 μg/mL for propiconazole exposed (n = 69) populations (Miller et al., 2002). We observed more than 12-fold increase in the EC_50_ values in the present dollar spot isolates compared to Miller et al. (2002) demonstrating the declining sensitivity of Georgian isolates to propiconazole over time. More importantly, propiconazole resistance was not observed by Miller et al. (2002) but was observed in 64.8% of isolates in 2022 in the current study. In addition, for thiophanate-methyl, a high difference in EC_50_ values between the less sensitive (LS: max 0.654 μg/mL) and the highly resistant (HR: min >1000 μg/mL) isolates was observed, while the sensitivity to propiconazole was more gradual in *Clarireedia* spp. population from Georgia with a negligible difference (0.003 μg/mL) between the sensitive (LS: max 0.098 μg/mL) and resistant (LR: min 0.101 μg/mL) groups. Previous studies have demonstrated that a single point mutation in the β-tubulin gene confers MBC fungicide resistance (Ostrander et al., 2014) and overexpression of either cytochrome P450-dependent sterol 14α-demethylase (*Cyp51*) gene or gain-of-function mutation in the *ShXDR1* were responsible for DMI fungicide resistance to *Clarireedia* spp. (Ma and Tredway, 2013; Sang et al., 2018).

We observed propiconazole-resistant isolates in the majority of Georgia’s counties while isolates resistant to thiophanate-methyl were concentrated in Spalding county demonstrating that thiophanate-methyl poses less risk and could still be applied judiciously in rotation with other fungicides. Two isolates (one each of *C. jacksonii* and *C. monteithiana*) collected from Spalding county of Georgia were multiple fungicide resistance (MFR) isolates, resistant to propiconazole and thiophanate-methyl. MFR occurs when an isolate or population becomes resistant to two or more fungicides from different chemical classes. MFR in *Clarireedia* spp. was initially described in 1983 with an isolate that exhibited resistance to thiophanate-methyl (MBC) and iprodione (DCF). Since then MFR has been reported in *Clarireedia* spp. populations from many regions across the US and poses an ongoing threat to the turf industry (Ok et al., 2011; Sang et al., 2019; Stephens and Kaminski, 2019). MFR isolates of propiconazole and thiophanate-methyl have been reported in Ohio (11.5%) (Jo et al., 2006), Tennessee and Northern Mississippi (10%) (Bishop et al., 2008), Wisconsin and Massachusetts (five out of seven populations of 1400 isolates) (Koch et al., 2009), and in the northeastern United States (13.5%) (Putman et al., 2010). In China (Hu et al., 2021) and the US (Salgado-Salazar et al., 2018), two or more *Clarireedia* species can co-exist on the same host type, leading to a challenging dollar spot management and suggesting that it is crucial to examine potential species-specific fungicide sensitivity patterns in these regions. Interestingly, in our study, Spalding county was the only county in Georgia where both species *C. jacksonii* and *C. monteithiana* were recovered and this county also held the MFR isolates. The higher level and frequent applications of fungicides to control this challenging disease in this county could explain the emergence of MFR strains. This fungicide resistance might have appeared independently in the two *Clarireedia* species as they were subjected to the same environmental conditions including exposure to fungicide, same geographic location, or even same host. Alternatively, the fungicide-resistant strains could have been introduced *via* migration from the northeast US. Further genetic analyses of pathogen populations will help to understand whether mutations or introductions events are responsible for the rise of fungicide-resistant isolates of *Clarireedia* spp. in Georgia.

Although effective chemical control options are available to manage dollar spot, increased public concerns about the use of synthetic pesticides have raised interest in biological control agents. In the present study, biofungicides *B. subtilis* QST713 and *B. amyloliquefaciens* F727 were as efficient as synthetic fungicides propiconazole and fludioxonil to suppress pathogen growth in laboratory tests. Further, the lowest label rate of synthetic fungicide propiconazole (1/4) was sufficient to entirely suppress *C. monteithiana* growth when mixed with *B. subtilis* QST713 (3/4). Similar to our findings, Marvin et al. (2020b) observed that ¼ label rate of *B. subtilis* QST713 exhibited the highest suppression in mycelial growth (>80%) over the ½ and full-strength treatments by establishing a containment zone around the inoculation plug that comprised short and stubby mycelium. Furthermore, in both the growth chamber and field experiments, we consistently observed the lowest disease severity and AUDPC with two different spray programs: a tank mix of 75% *B. subtilis* QST713 + 25% propiconazole applied every 28 days (T5) and 100% *B. subtilis* QST713 applied in rotation with a tank mix of 75% *B. subtilis* QST713 + 25% propiconazole every 14 days (T7). Program T5 could be cost-effective and would be recommended since it involves less number of fungicide applications and lower product volume to control dollar spot compared to program T7. If we merely consider the field-based study, our results showed that the application of *B. subtilis* QST713 every 7 days (T2), as well as the tank mix of 75% *B. subtilis* QST713 and 25% propiconazole applied in rotation with 100% *B. subtilis* QST713 every 14 days (T6) achieved the greatest reduction (>75%) in dollar spot severity. An acceptable turf quality (>7.0) was also obtained from programs T2 and T6, which further bolster the role of *B. subtilis* QST713 in integrated turfgrass management. More importantly, spray program T2 would be the most environmentally friendly since the program does not use any synthetic product and is solely based on biofungicide *B. subtilis* QST713. An economic analysis of the spray programs tested in this study would identify the most feasible and cost-effective programs. In a similar study by Marvin et al. (2020b), rotation applications of either pyraclostrobin or chlorothalonil (Daconil Ultrex™) with BCA at reduced label rates every 30 days suppressed dollar spot severity below 10% on a creeping bentgrass putting green at South Carolina despite the least efficacy of stand-alone biofungicide. Previous studies by Latin (2008) also agreed with our findings since reduced rates of chlorothalonil in a tank mix with BCAs *B. licheniformis* SB3086 and *B. subtilis* QST713 achieved similar control as the full labeled rate of synthetic fungicide alone when applied at a 7- or 14-day spray interval on creeping bentgrass in Indiana. Our results were also similar to Tomaso-Peterson (2006), where biofungicides *B. licheniformis* SB3086 and *Trichoderma harzianum* (TurfShield™) were found very effective in controlling dollar spot in Tifgreen Bermudagrass in Mississippi when applied alone (66-92% reduction) or alternated with chlorothalonil (85-95% reduction) at 7-, 14-, or 28-day interval without compromising the turfgrass quality. The tank mix of fungicides with different modes of action is usually recommended for better efficacy if the disease demands curative control. Nevertheless, turf managers who wanted to use the reduced label rate of synthetic fungicides or biofungicides as a stand-alone measure should monitor developing weather patterns with warm temperatures (15-30°C) and high humidity (>85%) since BCAs might lose residual efficacy and consistency in the context of excessively high disease pressure. A weather-based warning system developed recently using field data on relative humidity and temperature from Wisconsin and Oklahoma could be an important tool for implementing precision disease management strategies to control dollar spot (Smith et al., 2018). Based on the developed model, the research group revealed that fungicide loads could be curtailed by 30% while obtaining comparable disease control to the calendar-based spray program at a 20% spray threshold. Future research could be oriented on developing similar weather forecasting model for effective dollar spot management in the state of Georgia.

In a recent comprehensive review, Clarke et al. (2020) rated the efficacy of several fungicides and revealed that *B. subtilis* QST713 and *R. sachalinensis* extr. were not consistently effective in controlling dollar spot while other synthetic fungicides such as boscalid, propiconazole, fluxapyroxad, penthiopyrad, and thiophanate-methyl provided consistent, excellent disease control. Reports with low efficacy for full season control of dollar spot in creeping bentgrass have been found for commercial biofungicides products when tested against *C. jacksonii* (Koch et al., 2021). Nevertheless, the authors emphasized that additional research is indispensable to understand the role of different biofungicides. However, we observed a great efficacy of *B. subtilis* QST713 across *in vitro*, growth chamber, and field-based studies indicating its bright prospects. The differential level of disease pressure under which the efficacy of the biofungicides was rated in the previous studies could explain the discrepancy in the efficacy level. One of the main concerns with the incorporation of the BCA in the spray program was the need for consistent disease control over several years and under varying disease pressures. Interestingly, we found that the biofungicide spray programs worked consistently in controlling dollar spot in both seasons in the field despite different levels of disease severities at the beginning of the experiment (18.8 and 7.0% in the summer and fall season, respectively). In addition, although a high disease pressure (up to 45% severity in the non-treated control plot) was observed in our field experiments, *B. subtilis* QST713 stand-alone program (T2) was still efficacious. Further research could be carried out to answer questions on whether we can obtain consistent efficacy of *B. subtilis* QST713 in other warm-season turfgrasses infected with *C. monteithiana* and *C. jacksonii* in Georgia. From a pragmatic standpoint, turfgrass managers wanted to ensure acceptable levels of disease control from biological fungicides before they can utilize them in spray programs. Successful integration of biological fungicides into dollar spot management programs needs additional research to validate the efficacy of the products, especially at different levels of disease pressure. Nevertheless, the consensus of our findings with several previous studies holding promising results for BCAs will provide an impetus for effective dollar spot management to the turf industry (Latin, 2008; Marvin et al., 2020a; Koch et al., 2021b, Tomaso-Peterson, 2006).

Overall, our results from both growth chamber and field experiments suggested that an acceptable level of dollar spot control can be achieved even with a reduced dose of synthetic fungicide (propiconazole) when mixed with biofungicide (*B. subtilis* QST713). Furthermore, stand-alone use of biofungicide displayed comparable or even higher efficacy in controlling *Clarireedia* than synthetic fungicide alone, showcasing the potential of this biofungicide to curtail heavy reliance on chemicals in the field. The use of biofungicide will not only assure efficient disease control but also lower the risk of reduced fungicide sensitivity to propiconazole and thiophanate-methyl in *Clarireedia*, which is recently an emerging problem in the state of Georgia. To conclude, continuous surveillance of *Clarireedia* populations for fungicide sensitivities and the use of biofungicides in rotation and/or tank mixed with synthetic fungicides should provide an integrated approach to manage dollar spot on warm-season grasses in the southeast US.

## Data availability statement

The original contributions presented in the study are included in the article/**Supplementary Material**. Further inquiries can be directed to the corresponding author.

## Author contributions

BG and MA respectively wrote the major sections on biofungicide efficacy and fungicide sensitivity of the manuscript. BG designed the overall outline and prepared the first draft of the manuscript. SRC, WS, and CV helped on collecting data in the laboratory, growth chamber, and field experiments. BB, AM-E, and JB designed the research project and, provided guidance, critical suggestions, and feedback during the project execution and on the overall content throughout the manuscript preparation. All authors read, revised, and approved the submitted version.
