# Supplementary material for: Sensitivity of Clarireedia spp. to benzimidazoles and dimethyl inhibitors fungicides and efficacy of biofungicides on dollar spot of warm season turfgrass
Source: Front Plant Sci. 2023 Jun 9;14:1155670. doi: 10.3389/fpls.2023.1155670 (PMC10288879; doi:10.3389/fpls.2023.1155670)
Supplement: Supplementary file 1 [file DataSheet_1.docx]

# Supplementary Figures


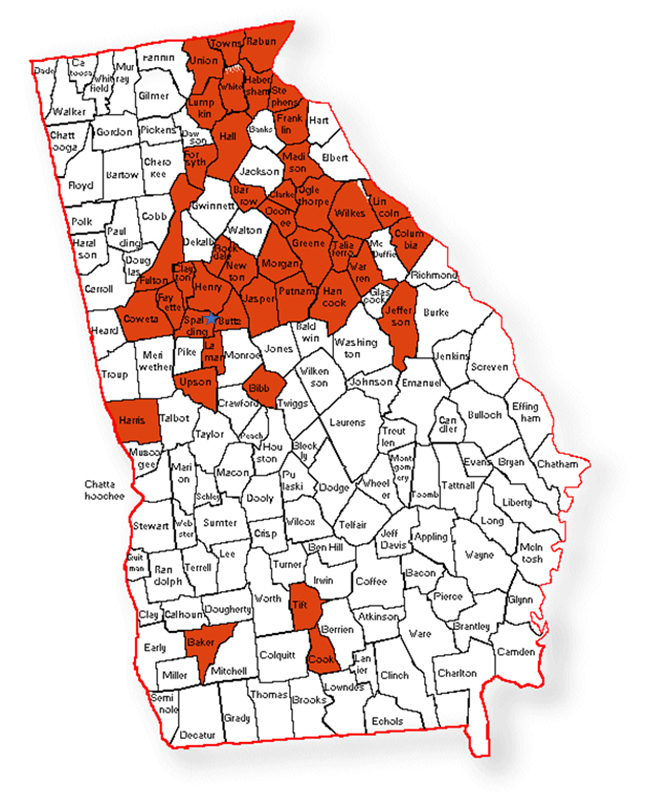


Figure S1: Map of Georgia showing *Clarireedia* spp. isolates collection counties marked in red color. Counties with asterisk consisted of double resistant isolates to both thiophanate-methyl and propiconazole.


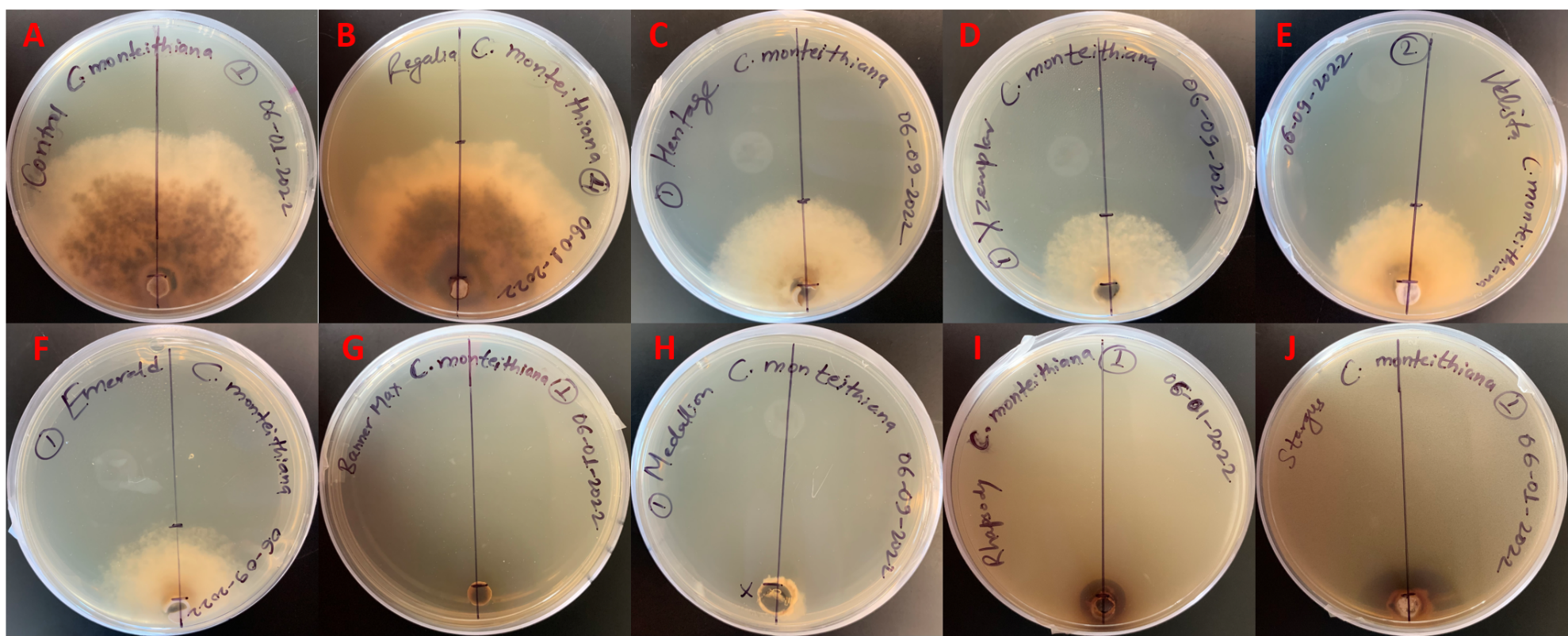


**Figure S2:** *In vitro* growth of *C. monteithiana* in fungicide-amended potato dextrose agar (PDA) plate after four days of incubation; control with no fungicide (A), *Reynoutria sachalinensis* extr. (Regalia) (B), azoxystrobin (Heritage) (C), fluxapyroxad (Xzemplar) (D), penthiopyrad (Velista) (E), boscalid (Emerald) (F), propiconazole (Banner Maxx) (G), fludioxonil (Medallion) (H), *Bacillus subtilis* QST713 (Rhapsody) (I), and *Bacillus amyloliquefaciens* F727 (Stargus) (J).


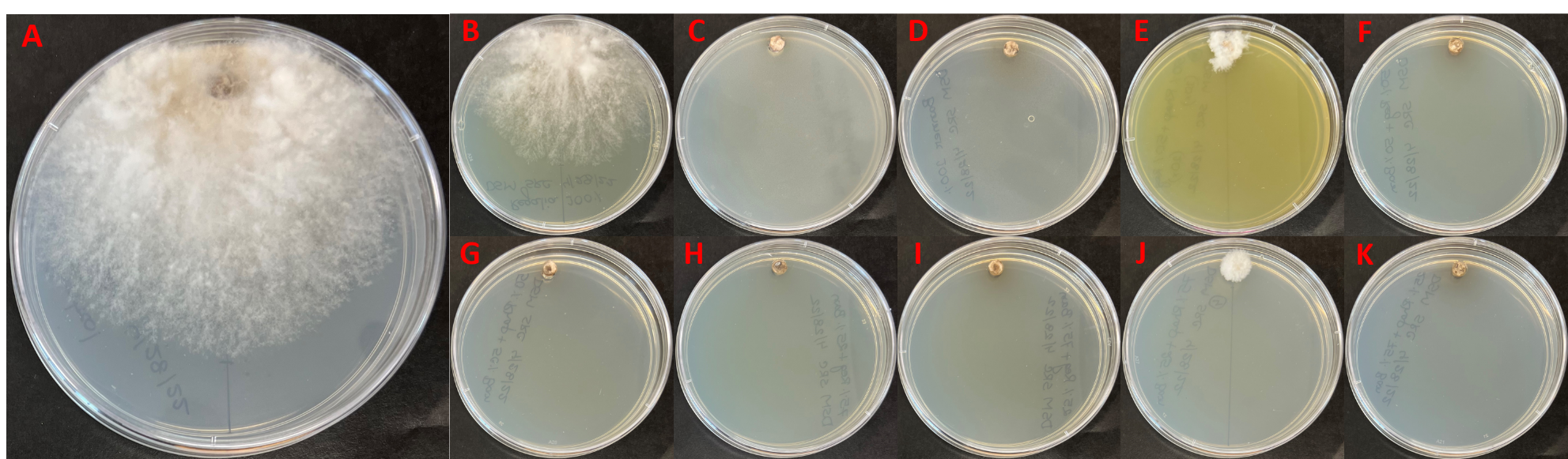


**Figure S3:** *In vitro* growth of *C. monteithiana* in fungicide-amended potato dextrose agar (PDA) plate after four days of incubation; control with no fungicide (A), 100% *Reynoutria sachalinensis* extr. (B), 100% *Bacillus subtilis* QST713 (C), 100% propiconazole (D), 50% *Bacillus subtilis* QST713 + 50% *Reynoutria sachalinensis* extr. (E), 50% *Reynoutria sachalinensis* extr. + 50% propiconazole (F), 50% *Bacillus subtilis* QST713 + 50% propiconazole (G), 75% *Reynoutria sachalinensis* extr. + 25% propiconazole (H), 25% *Reynoutria sachalinensis* extr. + 75% propiconazole (I), 75% *Bacillus subtilis* QST713 + 25% propiconazole (J), and 25% *Bacillus subtilis* QST713 + 75% propiconazole (K)


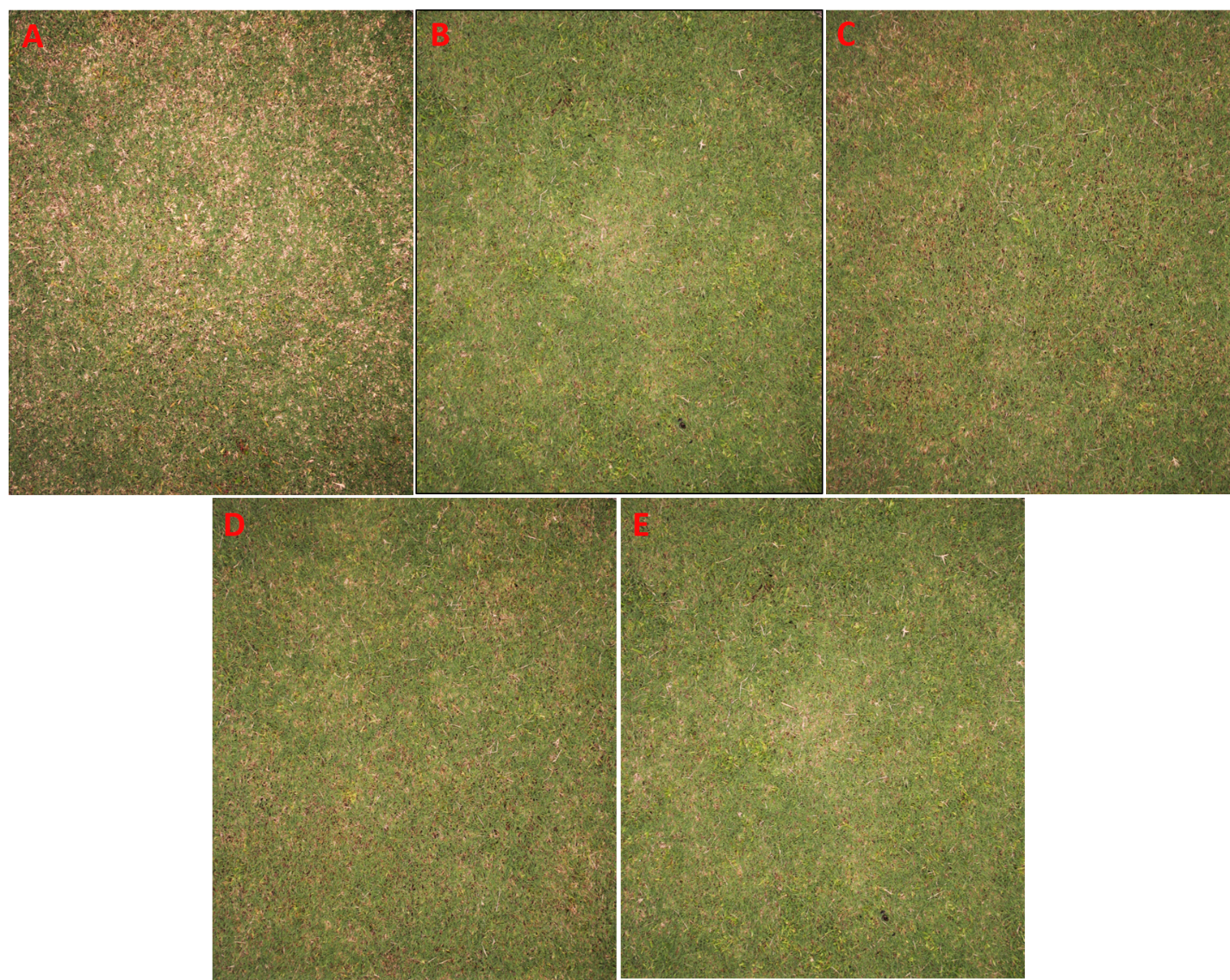


**Figure S4:** Experimental plots of non-treated control (T1) (A), *B. subtilis* QST713 applied every 7 days (T2) (B), a tank mix of 75% *B. subtilis* QST713 + 25% propiconazole applied every 28 days (T5) (C), 75% *B. subtilis* QST713 + 25% propiconazole tank mix in rotation with 100% *B. subtilis* QST713 applied every 14 days (T6) (D), and 100% *B. subtilis* QST713 in rotation with 75% *B. subtilis* QST713 + 25% propiconazole tank mix applied every 14 days (T7) (E)

(photo taken at 42 days after the start of the experiment at the UGA Griffin campus, Griffin GA during the summer season, 2022)
